# The relationship between school physical activity policy environment and physical literacy among primary school students: Evidence from Henan Province, China

**DOI:** 10.1371/journal.pone.0330991

**Published:** 2025-10-24

**Authors:** Hang Yin, Roxana Dev Omar Dev, Kim Geok Soh, Yajing Zhang, Zhiyuan Tan, Menglong Lian

**Affiliations:** 1 Department of Sports Studies, Faculty of Educational Studies, Universiti Putra Malaysia, Serdang, Malaysia; 2 Huaihe Road Primary School, Zhengzhou City, Henan Province, China; 3 Faculty of Sport and Physical Education, University of Belgrade, Belgrade, Serbia; 4 Zhengzhou Vocational College of Automobile Engineering, Zhengzhou, Henan Province, China; Neighborhood Physical Therapy, UNITED STATES OF AMERICA

## Abstract

**Background:**

Chinese primary school students spend the majority of their day at school, making the school physical activity policy environment (SPAPE) crucial to their development of physical literacy (PL). However, research exploring the relationship between SPAPE and PL remains limited. This study aims to investigate the association between SPAPE and students’ PL levels.

**Methods:**

A total of 408 primary school students (206 boys and 202 girls) were included in the data analysis. The School Physical Activity Environment Questionnaire (SPAEQ) and the Canadian Assessment of Physical Literacy-Edition 2 (CAPL-2) were used to assess the policy environment and PL levels, respectively. Pearson correlation coefficients were calculated to explore the relationship between the policy environment and PL. Additionally, ANOVA and MANOVA analyses were conducted to examine the effects of age, gender, and their interaction on the relationship.

**Results:**

A significant positive correlation was found between SPAPE and PL, with boys (r = 0.59, p < 0.01) and girls (r = 0.48, p < 0.01) both showing moderate to strong associations. MANOVA results revealed significant gender differences for Daily Behavior (DB) (F (1, 406) = 14.24, p < 0.01, partial η² = .04) and Motivation and Confidence (MC) (F (1, 406) = 4.72, p < 0.05, partial η² = .01). Significant age differences were observed for MC (F (4, 403) = 5.68, p < 0.01, partial η² = .05) and Knowledge and Understanding (KU) (F (4, 403) = 8.57, p < 0.01, partial η² = .08). No significant effects of age, gender, or interaction were found in relation to SPAPE.

**Conclusion:**

This study is the first to explore the relationship between PL and SPAPE in Chinese primary school students. The results highlight the significant association between SPAPE and PL, with notable gender and age differences. These findings emphasize the importance of tailoring PA policies to account for demographic factors to effectively promote PL.

## Background

Physical literacy (PL) has emerged as a critical concept in the field of physical education (PE) and health promotion, encompassing the motivation, confidence, physical competence, knowledge, and understanding necessary to engage in physical activity (PA) throughout life [1]. Research has consistently demonstrated that higher levels of PL are associated with increased participation in PA, improved physical health, and enhanced psychological well-being among children and adolescents [2–4]. However, the development of PL is not merely an individual responsibility; it is heavily influenced by environmental factors, particularly the school environment where children spend a significant portion of their formative years.

The school physical activity policy environment (SPAPE) plays a pivotal role in shaping children’s PA behaviors and their acquisition of PL [5,6]. The policy environment refers to the formal and informal rules, guidelines, and initiatives that govern PA opportunities in schools, such as mandatory PE classes, extracurricular sports programs, and activity-friendly schedules [7]. While previous studies have highlighted the individual impact of these environmental factors on PA levels [8,9], their specific influence on the multidimensional construct of PL remains under-explored. There remains limited attention paid to how specific aspects of the school environment influence various aspects of PL, such as motivation and confidence, physical competence, and knowledge and understanding. For example, policies designed to increase daily PA may not uniformly benefit all dimensions of PL; they may enhance students’ motivation to participate in activities while having a limited impact on their physical competence or understanding of movement skills. Similarly, material environments rich in resources and facilities might improve students’ physical competence but may not necessarily foster their confidence or motivation. These gaps in knowledge highlight the need for comprehensive studies that disentangle the unique contributions of school policy and material environments to the development of each PL dimension.

Furthermore, contextual factors such as gender and age may moderate the relationship between the school environment and PL. Boys and girls may perceive and respond to school policies and material environments differently due to societal norms, preferences, or expectations surrounding PA [10]. Likewise, younger and older children may have distinct developmental needs and capabilities that shape their experiences within the school environment. Understanding these differences is essential for designing tailored interventions that promote PL across diverse student populations.

This study aims to address these gaps by examining the relationships between the school policy environment, material environment, and the four dimensions of PL: daily behavior, knowledge and understanding, physical competence, and motivation and confidence. Specifically, it seeks to answer the following questions: (1) How do school policy environments influence the different dimensions of PL? (2) Are these relationships moderated by gender and age? The findings will contribute to a deeper understanding of how schools can optimize their environments to foster holistic PL development in children. This study will be the first to explore the relationship between the school policy environment and the PL of primary school students in China.

## Method

### Study design

This study employed a cross-sectional research design. The perceived levels of the policy and material environment were measured using the School Physical Activity Environment Questionnaire (SPAEQ) [11], and participants’ PL was assessed using the Canadian Assessment of Physical Literacy-Edition 2 (CAPL-2) [12]. Pearson’s product-moment correlation and multiple linear regression analyses were conducted to examine the relationships between the policy environment and PL. Additionally, the questionnaire included demographic information such as participants’ gender and age. Ethical approval for the study was obtained from the Ethics Committee of Universiti Putra Malaysia.

### Participants

Participants were recruited from five public schools in Henan Province, China, using a random sampling method. A total of 420 primary school students were selected for participation. Written informed consent was obtained from the parents or legal guardians of the participants prior to testing. The selected public schools had similar characteristics, including school size, curriculum structure, and institutional policies. Given Henan’s large population and substantial economic scale, the sample is representative and reflects the general population of primary schools across much of China.

### Measures

The perceived school physical activity policy environment of primary school students was assessed using the School Physical Activity Environment Questionnaire [11]. This questionnaire, developed by Chinese scholars, is tailored to the Chinese cultural context and has demonstrated good reliability and validity. The policy environment includes two dimensions: educational policy (EP) and policy implementation (PI). The questionnaire items were answered using a 5-point Likert scale, ranging from 0 (strongly disagree) to 5 (strongly agree). Reliability testing showed that Cronbach’s alpha coefficient was 0.87, confirming the internal consistency of the scale. Detailed information on the psychometric properties of the questionnaire is provided in Supplementary S1 File.

The PL levels of primary school students were assessed using the CAPL-2 (Canadian Assessment of Physical Literacy-Edition 2). The CAPL-2 is a comprehensive, scientifically grounded protocol that accurately evaluates children’s PL [13]. It assesses four dimensions of PL: Daily Behavior (30 points), Physical Competence (30 points), Motivation and Confidence (30 points), and Knowledge and Understanding (10 points), with a total maximum score of 100 points. The Daily Behavior dimension is assessed through self-reports of moderate-to-vigorous physical activity (MVPA) and objective measurement using accelerometers (7 days). Physical Competence is evaluated through the PACER (Progressive Aerobic Cardiovascular Endurance Run), plank, and CAMSA (Canadian Agility and Movement Skill Assessment), each contributing 10 points. Motivation and Confidence, as well as Knowledge and Understanding, are measured through questionnaires. The results of the confirmatory factor analysis (CFA) indicated that the model demonstrated a good fit (Chi-square = 2.28, p), confirming the scale’s validity. Reliability testing showed Cronbach’s alpha coefficient of 0.76, indicating excellent internal consistency and reliability of the assessment tool.

The data collection process started on October 15, 2024, and lasted for one week. This process was conducted by research assistants (one male and one female) in collaboration with the school’s PE teachers. Prior to data collection, the teachers underwent training to ensure they fully understood the assessment content and adhered to standardized scoring criteria. Additionally, informed consent was obtained from the participants’ parents before the data collection commenced. Pedometers were distributed to the children to track their step count over a seven-day period. Before the physical competence tests, the research assistants provided an oral explanation of the test procedures and demonstrated each task to ensure that the children comprehended the requirements. During the physical competence testing, participants were informed that they could withdraw from the test at any time if they felt any physical discomfort. The questionnaire data were collected during students’ study periods or after lunch, minimizing disruptions to their regular routines.

### Data analysis

All tests must be completed thoroughly before the data can qualify for the analysis phase. Data analysis was conducted using SPSS version 21. Prior to conducting the analysis, normality tests were performed on the data to ensure the reliability and validity of the results. Pearson’s correlation coefficient was used to examine the relationship between the policy environment and physical literacy. To examine group differences, MANOVA was conducted separately for two sets of variables: SPAPE, including EP and PI; and PL, including DB, MC, KU, and PC. These two MANOVAs were performed independently because SPAPE and PL are conceptually distinct constructs—representing environmental context and individual capability, respectively—and combining them into a single MANOVA would reduce theoretical clarity and potentially inflate multicollinearity. In addition, separate ANOVAs were conducted for the total scores of SPAPE and PL to explore group differences at the overall construct level. These composite scores were not included in the MANOVA analyses to avoid redundancy and overlapping variance with their respective sub-dimensions, which could distort the multivariate model. Partial eta squared (partial η²) was reported as a measure of effect size for ANOVA and MANOVA analyses to indicate the proportion of variance explained by each factor while controlling for other variables. According to Cohen’s guidelines, values of.01,.06, and.14 represent small, medium, and large effects, respectively [14].

## Result

Table 1 summarizes the descriptive statistics for SPASE and PL domains by gender and age. Boys scored higher than girls in DB and MC, while girls slightly outperformed in PC. PL increased with age, peaking at 12 years. SPAPE scores were similar across gender and age, with an overall average of 24.2 (5.1).

**Table 1:** Descriptive statistics for domain of SPAPE and PL.

Variable	Gender				Age (years old)										Total	
	Boys		Girls		8		9		10		11		12			
N	206		202		61		70		85		95		97		408	
	Mean	SD	Mean	SD	Mean	SD	Mean	SD	Mean	SD	Mean	SD	Mean	SD	Mean	SD
DB (0-30)	13.7	6.4	11.8	5.6	13.7	5.5	13.7	6.5	12.5	5.9	11.5	6.7	13.1	5.5	12.8	6.1
PC (0-30)	16.1	6.7	16.9	6.6	16.1	6.4	15.6	6.2	16.6	6.6	14.3	6.3	19.6	6.4	16.5	6.6
KU (0-10)	3.8	2.9	3.6	2.5	3.3	2.5	3.5	2.7	3.5	2.6	4.1	2.7	4.1	2.0	3.7	2.7
MC (0-30)	20.9	5.1	20.1	4.2	19.5	4.9	22.1	3.9	21.5	4.2	9.3	5.2	20.8	4.4	20.5	4.7
EP (3-15)	9.9	2.2	10.0	2.2	10.3	2.3	10.0	2.3	9.6	2.1	9.9	2.2	10.2	2.1	9.9	2.2
PI (4-20)	14.2	3.4	14.2	3.2	14.3	3.3	13.7	3.2	13.8	3.2	14.5	3.4	14.4	3.3	14.2	3.3
SPAPE (7-35)	24.2	5.2	24.2	4.9	24.6	5.3	23.7	5.3	23.4	4.6	24.5	5.2	24.6	4.9	24.2	5.1
CAPL-2 (0-100)	54.6	15.7	52.3	13.9	55.1	13.2	55.0	14.9	52.0	14.5	49.2	16.8	57.1	13.1	53.5	14.9

Note: DB, Daily Behavior; PC, Physical Competence; KU, Knowledge and Understanding; MC, Motivation and Confidence; EP, Educational Policy; PI, Policy Implementation; SPAPE, School Physical Activity Policy Environment; PL measured by CAPL-2.

Overall, there was a significant correlation between SPAPE and their levels of PL (boys: r = .59, *p* < .01; girls: r = .48, *p* < .01). Among boys, the correlation between KU and PC was significant but relatively weak (r = 0.18, *p* < .05), and it was the weakest among all statistically significant relationship. In contrast, no significant correlation between KU and PC was found in girls. For both boys and girls, the domains of EP, PI, and overall SPAPE showed the lowest correlations with PC. Aside from these findings, boys and girls demonstrated low to high correlations across all other domains, with correlation coefficients ranging from r = 0.29 to 0.96. The detailed content can be found in Table 2.

**Table 2:** Pearson’s correlation for domains of SPAPE and PL.

Variable	(1)	(2)	(3)	(4)	(5)	(6)	(7)	(8)
(1) DB								
Boys	–							
Girls	–							
(2) PC								
Boys	.39^**^	–						
Girls	.42^**^	–						
(3) KU								
Boys	.35^**^	.18^*^	–					
Girls	.34^**^	.14	–					
(4) MC								
Boys	.60^**^	.39^**^	.21^**^	–				
Girls	.57^**^	.31^**^	.27^**^	–				
(5) EP								
Boys	.44^**^	.27^**^	.41^**^	.44^**^	–			
Girls	.43^**^	.15^**^	.34^**^	.39^**^	–			
(6) PI								
Boys	.49^**^	.29^**^	.45^**^	.49^**^	.71^**^	–		
Girls	.47^**^	.21^**^	.21^**^	.38^*^	.64^**^	–		
(7) SPAPE								
Boys	.50^**^	.30^**^	.47^**^	.51^**^	.89^**^	.96^**^	–	
Girls	.49^**^	.21^**^	.29^**^	.42^**^	.87^**^	.94^**^	–	
(8) CAPL-2								
Boys	.84^**^	.75^**^	.47^**^	.78^**^	.52^**^	.57^**^	.59^**^	–
Girls	.84^**^	.76^**^	.47^**^	.73^**^	.42^**^	.44^**^	.48^**^	–

Note: DB, Daily Behavior; PC, Physical Competence; KU, Knowledge and Understanding; MC, Motivation and Confidence; EP, Educational Policy; PI, Policy Implementation; SPAPE, School Physical Activity Policy Environment; CAPL-2, PL measured by CAPL-2.

* , *p* < .05; ** , *p* < .01 (two-tailed).

As shown in Table 3, results revealed significant gender differences in DB, F (1, 406) = 14.24, p < .01, partial η² = .04, and MC, F (1, 406) = 4.72, p < .05, partial η² = .01, with boys showing higher scores in both domains. Significant age effects were observed for MC (F (4, 403) = 5.68, p < .01, partial η² = .05) and KU, F (4, 403) = 8.57, p < .01, partial η² = .08, with older students performing better. No significant gender × age interactions were found for any PL domains. ANOVA results indicated a significant main effect of age on overall PL F (4, 403) = 4.24, p < .05, partial η² = .04, while the effect of gender was not statistically significant F (1, 406) = 3.80, p > .05, partial η² = .01. For SPAPE domains (as shown in Table 4), MANOVA revealed no significant gender differences in either EP, F (1, 406) =.03, p > .05, partial η² = .00, or PI, F (1, 406) =.02, p > .05, partial η² = .00. Age differences were also non-significant for both EP, F (4, 403) = 1.28, p > .05, partial η² = .01 and PI, F (4, 403) =.86, p > .05, partial η² = .01. No significant gender × age interactions were found. Similarly, ANOVA showed no significant effects of gender, F (1, 406) =.00, p > .05, partial η² = .00, or age, F (4, 403) =.91, p > .05, partial η² = .01 on overall SPAPE scores.

**Table 3:** F value of different domains of PL by age and gender group.

	F_gender_	partial η²	F_age_	partial η²	F_gender×age_	partial η²
*MANOVA*						
DB	14.24^**^	.04	2.48^*^	.02	1.90	.02
MC	4.72^*^	.01	5.68^**^	.05	1.91	.02
PC	.07	.00	1.40	.01	1.08	.01
KU	.56	.00	8.57^**^	.08	1.18	.01
*ANOVA*						
PL	3.80	.01	4.24*	.04	1.66	.02

Note: ^*^, *p* < .05; ^**^, *p* < .01.

**Table 4:** F value of different domains of SPAPE by age and gender group.

	F_gender_	partial η²	F_age_	partial η²	F_gender×age_	partial η²
*MANOVA*						
EP	.03	.00	1.28	.01	.19	.00
PI	.02	.00	.86	.01	1.21	.01
*ANOVA*						
SPAPE	.00	.00	.91	.01	.73	.01

Note: ^*^, *p* < .05; ^**^, *p* < .01.

## Discussion

This study is the first to explore the relationship between the SPAPE and PL among Chinese primary school students, while also examining differences by gender and age. The results demonstrated a significant correlation between SPAPE and PL. Furthermore, age-related differences in PL were observed, particularly in the domains of KU and MC, as well as gender differences in the dimension of DB. However, no significant gender or age differences were found in the SPAPE and subdomains. Additionally, no interaction effects (age × gender) were identified for either PL or SPAPE.

Previously, no studies had examined the relationship between SPAPE and PL. This study provides novel and compelling evidence of a significant positive correlation between these two constructs, suggesting that comprehensive, coherent, and well-implemented policies play a critical role in promoting students’ overall PL. Unlike studies that have focused solely on how school policies increase PA levels, our findings extend the understanding of how policy environments shape students’ deeper engagement with PA—specifically, their knowledge, motivation, and competence, which are the core dimensions of PL [15].

The mechanisms through which SPAPE influences PL are multifaceted. SPAPE, which includes but is not limited to policies on curriculum standards, access to quality facilities, teacher training, inclusive programming, monitoring systems, and motivational incentives [16], shapes the educational and social environment in which physical literacy develops. When such policies are clearly defined and consistently implemented, they do more than simply increase participation in PA—they foster a learning environment that helps students understand the value of movement, develop the skills to participate confidently and build intrinsic motivation for lifelong engagement [17]. In this way, policies act not just as facilitators of PA behavior, but as systemic levers that nurture the full spectrum of PL. Thus, the relationship between SPAPE and PL is not only significant but foundational, with policy shaping both the conditions for and the outcomes of PL development.

Many previous studies have explored the relative age effect (RAE) on PL, often finding no significant differences across age groups and RAE is not a necessary factor for evaluating PL [18,19]. However, this study reveals significant age-related differences in the domains of KU and MC, reflecting the absolute age effect. Younger students displayed comparatively lower scores, which can be attributed to their cognitive and psychosocial developmental stages. These stages include less advanced cognitive processing abilities and limited prior exposure to structured physical education [20–22]. These findings align with developmental theories that emphasize the progressive nature of cognitive and motivational maturity, suggesting that younger children may benefit significantly from targeted interventions aimed at fostering early positive attitudes and intrinsic motivation toward PA. Educational interventions explicitly tailored to developmental stages can thus substantially enhance the knowledge, motivation, and long-term engagement of younger students in PA [23–26].

The significant gender disparities identified in both the DB dimension and the MC dimension, with boys demonstrating higher levels of daily PA and greater motivation and confidence compared to girls, echo findings from previous research [18,27]. These disparities may stem from persistent socio-cultural norms, gender stereotypes, and differential societal encouragement, which potentially limit girls’ motivation and opportunity to engage actively in physical activities [28,29]. In particular, the higher motivation and confidence observed in boys could be linked to societal expectations that encourage boys to engage in physical activities and excel in sports, reinforcing their self-confidence and intrinsic motivation [10]. On the other hand, girls may face societal barriers and reduced opportunities, resulting in lower self-efficacy and motivation for PA [30,31]. Consequently, developing inclusive and gender-sensitive policies and practices specifically designed to engage and sustain female participation, such as offering diverse and appealing PA programs, promoting female role models in sports, and implementing targeted motivational campaigns, is crucial to overcoming these barriers and promoting equity in PA participation.

Unexpectedly, our study found no significant differences in SPAPE across gender and age, nor any significant interaction effects between these demographic variables for either SPAPE or PL. This finding suggests a standardized and uniform policy implementation approach across schools in the Chinese educational system, reflecting a centralized model of policy-making and consistent enforcement mechanisms. In contrast to the diversity of policy environments seen in other educational systems, which can lead to demographic differences in outcomes [32–35], the relatively uniform approach in China may contribute to a more consistent educational experience for students, regardless of gender or age.

While the observed uniformity in SPAPE scores may reflect a centrally guided policy environment, this interpretation assumes consistent implementation across schools. However, the current study did not assess policy implementation fidelity, that is, the extent to which schools have put the prescribed policies into practice as intended. In reality, there may be substantial variation in how policies are translated into school-level actions due to differences in local resources, administrative capacity, staff engagement, or monitoring mechanisms [36]. This unmeasured variability could potentially mask important discrepancies between policy presence and actual execution, thereby influencing the observed associations between SPAPE and physical literacy. Without evaluating fidelity, it is difficult to determine whether the uniformity in scores reflects truly consistent implementation or merely uniform policy documentation.

The lack of demographic variation could also be influenced by the overarching role of national policy frameworks, such as China’s emphasis on the promotion of PA in schools through broad and centralized directives. These policies may inadvertently create a more equalized experience across different schools and regions, limiting the scope for differences that could be observed between genders or age groups. Additionally, the national education system’s strong emphasis on ensuring equal access to resources and opportunities for all students may have led to a standardization in the PA opportunities provided, leaving little room for gender or age-related differences to emerge. However, this uniformity in policy implementation may not fully account for the local variations that could affect physical literacy outcomes, particularly in regions with distinct cultural, socio-economic, or infrastructural characteristics. Studies from international contexts, where policy implementation can vary greatly between regions, suggest that these local differences—whether in the availability of resources, the degree of policy enforcement, or the cultural context—can create significant disparities in student outcomes [37].

To effectively enhance PL among students, it is crucial that policies go beyond merely providing opportunities for PA; they must create an environment that actively supports and promotes the development of all dimensions of physical literacy—physical competence, motivation and confidence, and knowledge and understanding. Policies designed to foster PL should be multi-faceted, addressing not only the availability of PA but also the quality of educational experience, teacher training, and the creation of supportive social and physical environments.

One of the most effective ways to promote PL through policy is by ensuring that PE curricula are both comprehensive and inclusive [38]. A well-designed policy should mandate the integration of PL as a core element of PE classes, ensuring that students are not only engaging in physical activities but are also developing the knowledge and skills necessary to participate in various physical activities confidently and competently. This could include fostering an understanding of the long-term health benefits of PA, teaching fundamental movement skills, and promoting the positive psychological effects of being active. Furthermore, policies should encourage schools to use a variety of teaching strategies, such as project-based learning or experiential learning, to engage students in activities that cater to diverse interests and learning styles, thereby ensuring that all students have the opportunity to develop their PL to their fullest potential.

Teacher training is another key aspect of policies aimed at enhancing PL [39]. Policies should require that physical education teachers receive ongoing professional development, ensuring they are equipped with the knowledge, tools, and pedagogical skills to effectively teach PL. This can include training in the latest physical education strategies, approaches to fostering motivation and confidence in students, as well as methods to assess and track students’ physical literacy progress over time. Additionally, teachers should be encouraged to develop their own PL, serving as positive role models for their students and fostering a culture of lifelong PA [40]. Policies could also promote the recruitment and retention of specialized professionals, such as sports psychologists or PL experts, who can provide targeted support to both students and teachers.

Furthermore, creating a supportive social environment within schools is essential for enhancing PL [41]. Policies should encourage schools to foster a culture of inclusivity, where PA is celebrated as an essential part of the school experience, regardless of gender, age, or physical ability. Encouraging peer support, creating opportunities for collaborative PA, and involving families and communities in promoting PA are all essential components of a comprehensive policy framework. Schools should also ensure that PA is accessible to all students, offering a wide range of extracurricular activities, sports clubs, and after-school programs that cater to various interests and abilities, and removing financial or logistical barriers to participation. Additionally, policies should prioritize the development of high-quality physical spaces. Adequate access to well-maintained sports facilities, playgrounds, and recreational areas is essential for facilitating daily PA [42]. Policies should advocate for investment in school infrastructure that supports diverse PA, including spaces for individual fitness, group sports, and informal recreational play. Such facilities not only provide the physical environment for engaging in activity but also help reinforce the message that PA is an important and enjoyable part of life.

Finally, policies should include robust monitoring and evaluation mechanisms to assess the effectiveness of PA programs and initiatives. By continuously evaluating the impact of policies on student engagement, motivation, and PL outcomes, policymakers can make data-driven adjustments to ensure that policies remain relevant, effective, and responsive to the needs of students. This approach helps create a dynamic system of policy improvement, where ongoing feedback informs future policy decisions and ensures that schools are equipped to meet the evolving needs of students in terms of PL development.

### Limitations

Although some findings and insights are discussed above, there are still some limitations in this study. Firstly, this study is a cross-sectional design, which restricts the ability to draw causal conclusions. Despite significant correlations being found between SPAPE and PL, the study cannot definitively establish the directionality of these relationships. Second, the study was conducted in a single province in China, which may limit the generalizability of the findings to other regions with different socio-economic, cultural, and educational contexts. China is a vast country with considerable regional diversity, and regional variations in policy implementation, resource allocation, and cultural attitudes toward PA could influence the outcomes. Third, some data relied on self-reported from students, which can be subject to biases, including social desirability bias and recall bias. Lastly, our study did not assess the fidelity of policy implementation at the school level. Differences in how policies are enacted and enforced within individual schools may still exist and could influence their effectiveness.

### Future direction

Building upon the current findings, several directions are recommended for future research. To better understand the causal mechanisms linking school physical activity policy environments and physical literacy, longitudinal or experimental designs should be employed. These approaches would help clarify the temporal sequence and underlying processes of the observed associations. In addition, future studies should expand the geographic scope beyond a single province to include more diverse regions or a nationally representative sample. This would allow for the examination of regional differences in policy contexts and enhance the generalizability of findings. Moreover, the use of mixed-method approaches may help reduce biases and enrich the interpretation of students’ physical literacy. Finally, future research should incorporate evaluations of policy implementation fidelity at the school level, as variability in execution may significantly influence the effectiveness of policy environments on student outcomes.

## Conclusion

This study provides pioneering empirical evidence on the relationship between SPAPE and PL among Chinese primary school students, which demonstrates a significant positive correlation between SPAPE and PL. The findings emphasize the need for age-appropriate and gender-sensitive policies that promote equal access to PA opportunities and support students’ motivation, knowledge, and competence. Despite the uniformity of policy implementation observed across schools, the study calls for further research into regional variations and the fidelity of policy implementation. Overall, this research provides valuable insights for improving policies to enhance PL and contribute to students’ lifelong engagement in PA.

## Supporting information

S1 File Psychometric properties of SPAPE. (DOCX)

S2 File Inclusivity-in-global-research-questionnaire. (DOCX)
