# Supplementary material for: The relationship between school physical activity policy environment and physical literacy among primary school students: Evidence from Henan Province, China
Source: PLoS One. 2025 Oct 24;20(10):e0330991. doi: 10.1371/journal.pone.0330991 (PMC12551888; doi:10.1371/journal.pone.0330991)
Supplement: S1 File — (DOCX) [file pone.0330991.s001.docx]

**1. Overview**

This study used the Policy Environment dimension of the School Physical Activity Environment Questionnaire (SPAEQ) [1], focusing specifically on two subdimensions: Educational Policy (EP) and Policy Implementation (PI). The adapted instrument was tested among Chinese primary school students and validated for use in this population.

**2. Factor Structure and validity coefficients**

| Subdimension | Number of items | Item example | Validation Method |
| --- | --- | --- | --- |
| EP | 3 | *The physical fitness exam for primary school graduates motivated me to participate in physical activities* | CFA |
| PI | 4 | *Our school has well-organized recess activities every day* | CFA |


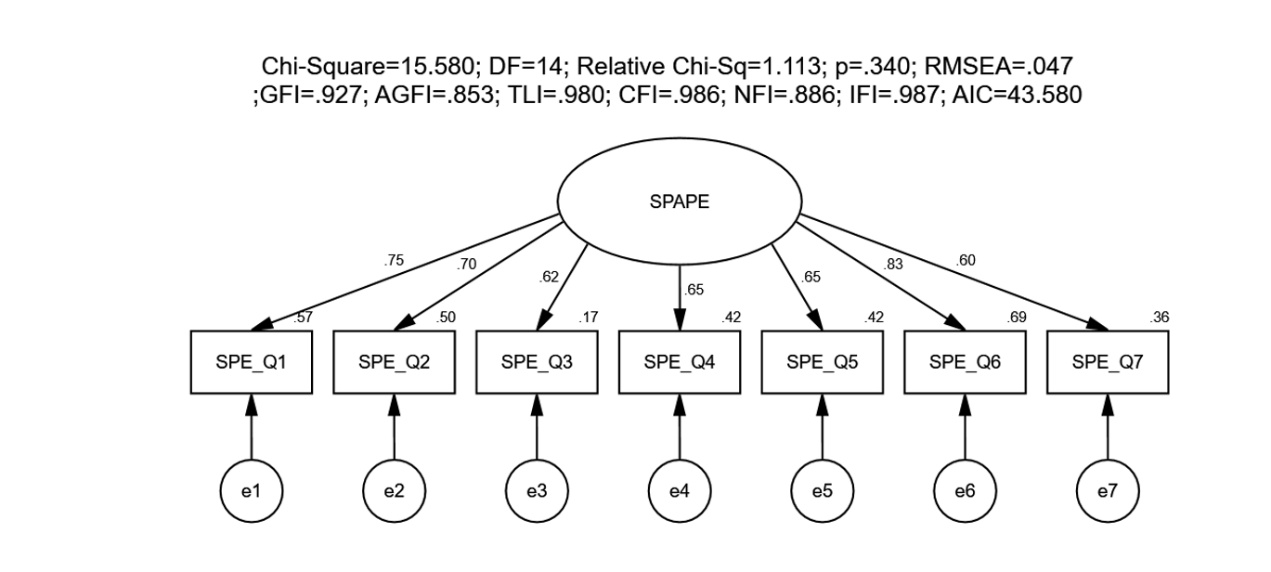


**3. Response Format and Scoring**

- **Scale**: 5-point Likert scale (1 = Strongly Disagree, 5 = Strongly Agree)
- **Scoring**: Average scores were calculated for each subdimension and the overall policy environment score.

**4. Reference:**

Guo Kelei. Study on the Relationship Between Physical Activity, Environment, Exercise Intentions and Physical Activity of Junior High School Students. Doctoral thesis, Shanghai University of Sport. 2019. doi:10.27315/d.cnki.gstyx.2019.000018
